# Haemoglobin for Fall Risk Screening in Gynaecological and Obstetric Wards: Retrospective Survey and Delphi Validation

**DOI:** 10.1002/nop2.70124

**Published:** 2024-12-26

**Authors:** Bijun Mao, Yan Chen, Chunsheng Wang, Yihan Ma, Huifeng Gu, Ya Shen, Luping Liu, Peihong Zhou, Huiping Jiang

**Affiliations:** ^1^ Nursing Department Huzhou Maternity & Child Health Care Hospital Huzhou China; ^2^ School of Medicine Huzhou Teachers College School Huzhou China; ^3^ Lee Shau Kee School of Business and Administration Hong Kong Metropolitan University Hong Kong China

**Keywords:** anaemia, Delphi surveys, fall, haemoglobin, nursing, safety

## Abstract

**Aims:**

The objective of this study is to ascertain the suitability of haemoglobin as a screening factor for falls among obstetrics and gynaecology inpatients and to formulate a stratified scheme for assessing fall risk based on haemoglobin.

**Design:**

A retrospective analysis and Delphi surveys were employed for this investigation.

**Methods:**

Initially, a retrospective survey analysed falls among obstetrics and gynaecology inpatients in two hospitals from January 1, 2020, to July 10, 2022. Descriptive statistics, receiver operating characteristic (ROC) curve analysis, Youden index, sensitivity and specificity were utilised for data examination. The conclusions drawn were subsequently validated by Delphi surveys, featuring 21 experts participating in five rounds of consultation. The Kappa value and the coefficient of variation (CV) were employed to assess expert advice.

**Results:**

The area under the Receiver Operating Characteristic curve (AUC) of haemoglobin was 0.762 ± 0.030, 95% CI (0.703, 0.821). The highest Youden index was 0.425, with sensitivity at 0.705 and specificity at 0.720 when haemoglobin was 107.5 g/L. Two consensuses were reached by experts: anaemia was important in causing falls in obstetrics and gynaecology wards, and haemoglobin should be employed as a screening factor for falls. The stratification of anaemia was developed as follows: ≥ 110; 90–109; 60–89; and < 60 g/L. Approval for the final results was unanimous among all experts. The Kappa value (K*) was 1, and the CV of expert advice ranged from 0.092 to 0.219.

**Conclusions:**

Haemoglobin could potentially be used as a predictor of fall risk in Gynaecological and Obstetric Wards. The recommended stratified scheme for anaemia in fall risk assessment is as follows: ≥ 110; 90–109; 60–89; and < 60 g/L.

**Implications for the Profession and/or Patient Care:**

- What problem did the study address?

The study revealed a relationship between falls and haemoglobin in obstetrics and gynaecology inpatients. It also proposed a stratification scheme for assessing fall risk based on haemoglobin levels.

- What were the main findings?

Haemoglobin has a good performance on fall risk prediction in Gynaecological and Obstetric Wards.

The stratified scheme of anaemia for fall risk assessment was suggested as follows: ≥ 110; 90–109; 60–89; and < 60 g/L.

- Where and on whom will the research have an impact?

Nurses and inpatients in obstetrics and gynaecology wards will be affected by the results of this study, and it provided a reference for fall prevention.

**Reporting Method:**

This study has adhered to relevant EQUATOR guidelines and named the reporting method.

No Patient or Public Contribution.

## 1 Introduction

Falls represent a prevalent and persistent challenge within healthcare settings and often result in severe consequences such as hip fractures, subdural hematoma and even fatalities (Burns et al. 2020). Notably patients who experience falls may extend their hospital stay by 6–12 days incurring additional costs ranging from $19,376 to $32,215 (Spetz, Brown, and Aydin 2015). Beyond the physical toll, falls adversely impact the quality of patients' lives and pose a significant financial burden on the healthcare industry (Burns et al. 2020). Consequently, falls serve as a critical benchmark for evaluating nursing quality. In the pursuit of patient safety, nurses concentrate on preventing falls, with risk reduction representing a focal point of prevention endeavours.

Predictably, falls necessitate preventive measures, prompting numerous studies to assess various fall prevention strategies. Evidence supports that many falls can be averted through appropriate assessment and intervention (Xing et al. 2023). Recognising patients at an elevated risk of falls or identifying modifiable risk factors is crucial for subsequent intervention. In care settings, a fall risk screen serves to identify individuals requiring a more comprehensive fall risk assessment and intervention.

The World Health Organisation's International Classification of Functioning, Disability, and Health (WHO‐ICF) aids in categorising fall risk factors (de Kleuver et al. 2021; Fehrmann et al. 2023; Story et al. 2021). Intrinsic factors include a history of falls/Age/Gender/Living alone/Ethnicity/Medicines/Medical conditions/Impaired mobility and gait/extremity disability/Sedentary behaviour/Psychological status/Nutritional deficiencies/Impaired cognition/Visual impairments/Foot problems. Extrinsic risks involve environmental hazards (poor lighting, slippery floors, uneven surfaces, etc.) footwear, clothing/inappropriate walking aids or assistive devices.

Many fall risk assessment tools have been developed based on these risk factors (Kuş, Büyükyılmaz, and Ardıç 2023). These tools constitute an integral part of fall prevention programs in care settings (Welch et al. 2021). By identifying fall‐risk patients, the tools help nurse intervene, reducing the incidence of falls, some have been evaluated for reliability and predictive validity in prospective studies and have reasonable sensitivity and specificity. Such as the MFS, St. Thomas Risk Assessment, the Hendrich II Fall Risk Model and so on (Kim et al. 2022). However, despite implementing various improvement strategies with these assessment tools, fall rates remain high in care settings, primarily in the elderly, but also among obstetrics and gynaecology patients. Some studies showed that gynaecological and obstetric patients were at a high fall risk (Fisher, Harmouche, and Kilic 2022). Notably, the characteristics of falls, related injuries and circumstances vary significantly across clinical departments (Mikos et al. 2021; Schwendimann et al. 2008). Therefore, hospitals should tailor fall prevention strategies, considering the unique characteristics associated with patient falls in different clinical departments.

Given the diverse factors contributing to falls, including disease‐related and treatment‐related factors, existing patient fall risk scales tend to address specific intrinsic and extrinsic factors. However, they may fall short of comprehensively assessing all fall risks, necessitating more patient‐centred risk assessments and screenings. Researchers propose integrating disease‐related factors into fall risk screening, allowing for more accurate identification of individuals at risk. Therefore, elucidating the factors associated with obstetric and gynaecological falls becomes imperative to mitigate their occurrence.

Given the diverse factors that contribute to falls, including disease‐related and treatment‐related factors, existing patient fall risk scales tend to address specific intrinsic and extrinsic factors. However, they may fall short in comprehensively assessing all fall risks, necessitating more patient‐centred risk assessments and screenings.(de Kleuver et al. 2021; Fehrmann et al. 2023; Story et al. 2021). Researchers propose integrating disease‐related factors into fall risk screening, allowing for more accurate identification of individuals at risk (Zhang et al. 2023). Therefore, elucidating the factors associated with obstetric and gynaecological falls becomes imperative to mitigate their occurrence.

Research by Andrew S. Artz highlights anaemia as a potent prognostic factor for elderly frailty‐related issues, including falls and mortality (Artz 2008). Additionally, patients with chronic kidney disease, a population prone to anaemia, face an elevated risk of falling (Kara et al. 2022). Dharmarajan's study demonstrated a 22% decrease in the risk of falls for every 1.0 g/dL increase in haemoglobin (*p <* 0.05) among anaemic patients, emphasising the intricate link between anaemia and fall risk (Dharmarajan, Avula, and Norkus 2007).

We wondered, does anaemia contribute to the risk of falls on obstetrics and gynaecology inpatients? Considering the various disorders leading to blood loss and anaemia in this patient population such as dysfunctional uterine bleeding, childbirth and so on since the effect of anaemia on falling varies according to haemoglobin content, how should anaemia be stratified? Thus, this study was designed to ascertain whether anaemia should be used as a screening factor for falls in obstetrics and gynaecology wards, and proposes a stratified scheme for assessing fall risk, ultimately, providing the basis for developing a quality management strategy to enhance the safety of women hospitalised in obstetric and gynaecology wards.

## 2 Materials and Methods

### 2.1 Aim and Study Design

A retrospective analysis was conducted to assess the impact of haemoglobin on fall risk prediction in gynaecological and obstetric wards and determined the optimal cut‐off point. Delphi surveys were conducted to establish a consensus on the suitability of haemoglobin as a screening factor for falls among obstetrics and gynaecology inpatients. Additionally, the surveys aimed to define a stratified scheme for haemoglobin in fall risk assessment.

### 2.2 Retrospective Survey

Haemoglobin levels were compared between falling and non‐falling patients in obstetrics and gynaecology wards to investigate the potential contribution of anaemia to falls. The analysis also aimed to determine the cut‐off point of haemoglobin as a fall risk screening factor.

### 2.3 Fall Definition

There were two fall states in our study. Fall: the individual was in a low‐level position due to the sudden and unconscious loss of posture without external forces; Pre‐fall: Without external forces, the patient would assume a low‐level position because of sudden body balance loss if someone didn't pro.

### 2.4 Sample Size

Adhering to the recommendations of Yung Hee Sung (Sung et al. 2014), who suggested 84 subjects for each group. We calculated the sample size with the formula: *n* = (*Zα*/*δ*)^2^ *p* × (1 − *p*) budget sample size. *α* = 0.05, *Zα* = 1.96, *δ* = 0.075. According to our pre‐test, sensitivity (pse = 0.75), specificity (psp = 0.78) and the sample size were calculated, respectively: 117 cases for the no‐fall group, and 128 for the fall group.

### 2.5 Setting and Participants

This study was conducted in an Obstetrics and Gynaecology hospital and a general hospital with eight obstetrical wards and seven gynaecological wards from January 1, 2020, to July 10, 2022. The average length of stay in these wards was 4–5 days.

Patients under 18 years of age were excluded from the research because (1) regulation for the protection of Human Subjects requires parental consent for the inclusion of minors in research and (2) falls by young children include falls from climbing and tripping which are a part of normal activity and thus are considered a separate phenomenon; patients who had been hospitalised in the wards before the study began were excluded too.

A total of 63,568 patients met the criteria, including 136 fallers and 63,432 no‐fallers. Fallers who met the criteria were included in the fall group. Each no‐faller was assigned a sequential number, and then randomly selected 120 patients from this group using SPSS for inclusion in the control group.

### 2.6 Data Collection

Data were collected by two registered nurses on the number of patients who fell during hospitalisation rather than the number of falls. The fall records were obtained from the hospital's adverse‐event reporting system. Demographic information was acquired upon admission, and clinical data were extracted from the hospital's electronic records. Missing values were imputed with the mean or modal values.

Regular fall risk assessments using the MFS were conducted by the participating hospitals to minimise patient falls. Haemoglobin values were collected at the closest time node to the fall for falling inpatients and at the highest Morse Fall Scale (MFS) score for non‐falling inpatients, as a higher MFS score implies an elevated risk of falling (Morse, Morse, and Tylko 1987; Morse et al. 1989).

### 2.7 Variables and Measurement

An integrative review of the literature identified clinical and epidemiological relevance criteria that were used to define the study variables. The following variables were conceptually and operationally defined in a previous study on fall risk factors (Hartley et al. 2023; Wapp et al. 2023): age, gender (male, female and transgender), length of hospital stay, time of fall, a fall attributed to physiological causes within the last three months, disorientation, confusion, agitation, insomnia, activity status, surgery (postoperative status during the current hospitalisation), visual acuity change and diagnosis.

### 2.8 Delphi Surveys

#### 2.8.1 Establishment of the Research Team

The research team comprised two nursing management experts, a university statistics teacher, a university student, a gynaecological specialist nurse and an obstetrics specialist nurse. The main tasks of the research team were: (1) to develop expert consultation questionnaires; (2) to select and contact advisory experts; and (3) to summarise expert opinions for processing and analysis. The authors declare that the members of the expert group are accountable for the consultation results, ensure that the feedback from the questionnaire originates from themselves and guarantee that the research content is not disclosed to other consulting experts.

#### 2.8.2 Recruit and Identification of the Advisory Experts

Expert recruitment employed a purposive approach, distributing flyers to major medical institutions in Huzhou city in the east of China, and an Obstetrics and Gynaecology Specialised Hospital affiliated with Zhejiang University. Selection criteria were stated in flyers: (1) clinical nursing experience in obstetrics and gynaecology for at least ten years, (2) a bachelor's or higher degree, a medium or above professional and technical position; and (3) willingness to participate in this study and the ability to continue participating until an expert consultation is completed. When potential participants expressed an interest in the study, the researcher contacted them by phone or WeChat and arranged a consultation. Twenty‐one expert consultants were enrolled in the study. Before the study, the investigator could not identify the participating experts and did not know the experts participating in the study.

### 2.9 Implementation

The research team made the questions into an electronic questionnaire. The experts were contacted directly by the research team members on WeChat and explained the purpose of the research to the experts in person and obtained their consent before the consultation started. All consultation questionnaires were done by Questionnaire Star (The questionnaire was obtained by scanning a QR code by mobile phone). The experts completed the questionnaires alone and were not able to see each other's responses. Questionnaires can only be submitted after all questions have been answered. Weekly reminders were sent via email to non‐responders to ensure the timely completion of tasks. All the questions in the questionnaire were reviewed by a teacher of nursing to Reduce Researcher bias. The degree of coordination of expert opinions is considered as high by the coefficient of variation (CV) < 0.15, and the CV of each round consultation below 0.15 can enter the next round, otherwise the consultation will be repeated until CV < 0.15. Five rounds of expert consultation were carried out in this study.

Round 1: Experts rated the importance of anaemia in causing falls and agreement with using haemoglobin as a screening factor for falls on a 5‐point Likert scale (1: Strongly Disagree; 5: Strongly Agree). Basic information about the experts and their familiarity and competence in various domains was also collected. Analysis of ratings, distribution of ratings, and mean and median values were calculated by the researchers.

Round 2: Experts were informed of the result of round 1 and asked to rate the items based on their ratings, the mean and median values and the distribution of ratings. The research team obtained the level of consensus.

Round 3: To develop a stratified scheme of haemoglobin in fall risk screening, the research team collaborated with clinical nursing experts, initially proposed stratified schemes and formed the questionnaire of round 3. Experts were presented with proposed schemes and asked to select the most reasonable one, and they were encouraged to make new recommendations.

Round 4: The results of round 3 were presented to the experts in the questionnaire. Schemes with low selection rates in the last round were eliminated to ensure that expert opinion was concentrated. Experts were asked to reconsider their choices after the stratified schemes had been modified based on their suggestions. This process continued until a scheme obtained agreement exceeded or closely approached 70% of the experts (García‐Vicuña et al. 2021).

Round 5: Experts were informed of the results of round 4 and asked to assess approvement of the final stratification scheme on a 5‐point Likert scale (1: Strongly Disagree; 5: Strongly Agree). Approval was considered achieved when recognition by experts exceeded or closely approached 70% (García‐Vicuña et al. 2021).

### 2.10 Data Analysis

The data were processed using SPSS for Windows ver 12.0.1. Descriptive statistics such as frequencies, percentages, means and standard deviations were used to analyse the demographic and clinical characteristics of the subjects. the relationships between the subjects and demographics and clinical characteristics were examined with the *χ* ^2^ and *t*‐tests. Moreover, the *t*‐test was adopted for the normal distribution, the Mann–Whitney *U* test was used for the non‐normal distribution and the chi‐square test aimed to compare the categorical data. The optimal cut‐off of haemoglobin was analysed with the gold standard of patients who fell while hospitalised by the area under the subject Receiver operating characteristic curve (AUC) and Youden index. The expert's authority coefficient (Cr) was determined. The Kappa value and the coefficient of variation (CV) were used to examine the level of coordination and concentration of expert advice. CV value was below 0.15 indicating a high coordination degree.

### 2.11 Ethical Considerations

Ethical approval was granted by the (REDACTED). In the Retrospective survey, all patients in this study were allocated a number of substitute identity information. Authors had no access to information that could identify individual participants during or after data collection. The data file was encrypted and accessed only by the main researchers. In the Delphi survey, participants were provided with an information sheet that explained the research and the nature of their participation. Consent was sought freely and potential participants were made aware that there would be no adverse outcomes for the individual should they choose not to participate or to withdraw at a later date.

### 2.12 Bias

In the retroactive survey, data collectors were trained, and data collection criteria were standardised. We included all patients who fell incidentally in the study to reduce selective bias. In the Delphi survey, anonymity ensured that experts were not subject to peer pressure during the Delphi surveys and that they could speak freely without influencing each other's opinions. In this study, an above 90% response rate was maintained both in terms of publication ethics and the anonymity inherent in the Delphi method.

## 3 Results

### 3.1 Demographic Characteristics of Subjects in Retrospective Survey

A total of 136 fallen inpatients and 120 no‐fallen inpatients were included in this study, contributing to 269,533 patient days in gynaecological and obstetric wards. The fall rate was 0.21% (0.504 days per 1000 patient days). Table 1 indicated whether or not the subjects fell based on their demographics. All participants were female, and falls in gynaecological and obstetric wards did not differ based on age, education, marriage, smoking and drinking.

**TABLE 1:** Comparisons of demographic characteristics between groups (*n* = 256, %).

Items		Fall	No‐fall	*x* ^2^ or *t*	*p*
Age		31.54 ± 11.111	31.25 ± 9.280	0.228	*0.820*
Education	Under the university	99 (50.77%)	96 (49.23%)	2.000	*0.177*
	University above	37 (60.66%)	24 (39.34%)		
Marriage	Divorcement	3	2	2.872	*0.455*
	The partner died	1	0		
	Unmarried	0	2		
	Married	132	116		
Smoking	Yes	1	2	0.477	*0.490*
	No	135	118		
Drinking	Yes	3	4	0.305	*0.581*
	No	133	116		

*Note:* There were no significant differences between the two groups in age, education, marriage, smoking, and drinking.

### 3.2 Clinical Characteristics of the Subjects

Reasons for hospitalisation included induced labours, preterm births, births, dysfunctional uterine bleeding, tumours, perineum hematomas, inflammatory diseases, prolapses and abortion. No visual disorders or paralysis and no falls in the previous three months due to physiological causes were observed. No disorientation and/or confusion. Surgery was associated with falls (*x* ^2^ = 7.306, *p* = 0.007). Table 2 shows whether the subjects fell according to their clinical characteristics.

**TABLE 2:** Comparisons of clinic characteristics between groups (*n* = 256, %).

Items		Fall		No‐fall		*x* ^2^ or *t*	*p*
		*n*	%	*n*	%		
Admission course	Emergency	32	55.17	26	44.83	0.126	0.722
	Outpatient admission	104	52.52	94	47.48		
Hospitalisation (days)	—	6.06 ± 3.133		6.16 ± 3.084		−0.255	0.799
Admission method	Walking	105	50.72	102	49.28	2.595	0.273
	Wheelchair	22	64.71	12	35.29		
	Stretcher car	9	60.00	6	40.00		
Activity status	Fully dependent	0	0	0	0	2.678	0.102
	Partially dependent	3	100	0	0		
	Independent	133	52.57	120	47.43		
Diabetes	Yes	20	62.50	12	37.50	1.291	0.256
	No	116	51.79	108	48.21		
Hypertension	Yes	20	62.50	12	37.50	1.291	0.256
	No	116	51.79	108	48.21		
Insomnia	Yes	8	80.00	2	20.00	3.018	0.082
	No	128	52.03	118	47.97		
Surgery	Yes	57	64.77	31	35.23	7.306	0.007*
	No	79	47.02	89	52.98		
Kidney disease	Yes	0	0	1	100	1.138	0.469
	No	136	53.33	119	46.67		
Hepatopathy	Yes	3	75.00	1	25.00	0.781	0.377
	No	133	52.78	119	47.22		
Agitation	Yes	0	0	1	100	1.138	0.469
	No	133	52.78	119	47.22		
Disease of immune system	Yes	3	75.00	1	25.00	0.781	0.377
	No	133	53.20	117	46.80		
Cardiac disease	Yes	4	50.00	4	50.00	0.032	0.857
	No	132	53.23	116	46.77		

* Fisher's precise probabilities.

### 3.3 Difference in Haemoglobin Between Groups

The haemoglobin levels in both groups followed normal distributions (*Z* = 0.717, *p* = 0.684; *Z* = 0.980, *p* = 0.292). In the fall group, haemoglobin levels ranged from 47 to 132 g/L, with a mean value of 96. 1 ± 22.4 g/L; in the no‐fall group, haemoglobin levels ranged from 63 to 150 g/L, with a mean value of 115.4 ± 16.7 g/L; there was a statistically difference between the mean haemoglobin levels of the two groups (*t* = 6.631, *p* = 0.000).

### 3.4 The ROC and the AUC of Haemoglobin

**FIGURE 1:**
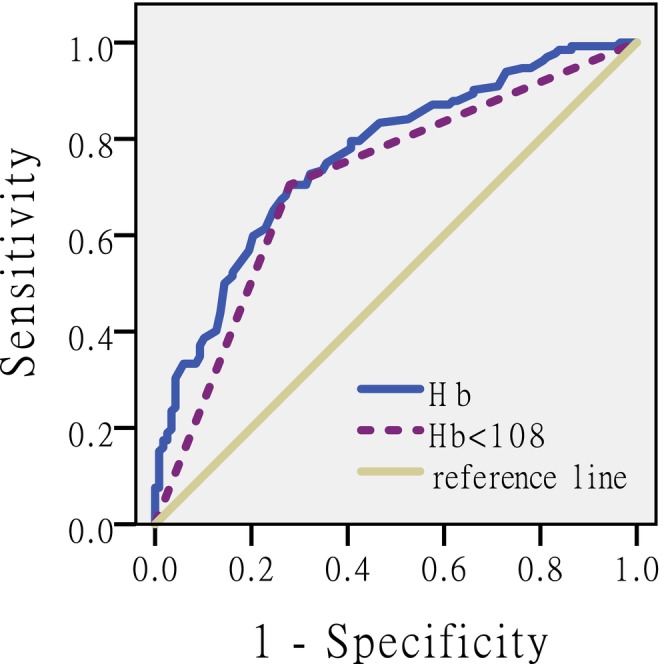
ROC curve of haemoglobin. ROC curve showing the capacity of haemoglobin in discriminating between the status of faller and non‐faller in a group of 256 Obstetrics and Gynaecology inpatients.

The ROC curve of haemoglobin illustrate its performance as a binary classifier model in predicting inpatient falls at different thresholds. Figure 1 illustrates the haemoglobin ROC curve. The closer the ROC curve is to the upper left, the higher the haemoglobin sensitivity to predict the risk of falls, the lower the missed diagnosis rate; the higher the specificity, the lower the misdiagnosis rate. We know that AUC > 0.5 indicates that this index has predictive value, and a larger AUC represents a higher predictive value. The AUC of haemoglobin was 0.762 ± 0.030, 95%CI (0.703, 0.821) (AUC > 0.5).

ROC curve showed the capacity of haemoglobin in discriminating between the status of faller and non‐faller in a group of 256 Obstetrics and Gynaecology inpatients.

### 3.5 Sensitivity, Specificity, Youden Index and AUC of Haemoglobin

Table 3 presents the discrimination test results based on haemoglobin levels. With increasing haemoglobin levels, sensitivity decreased while specificity increased. At a haemoglobin level of 107.5 g/L, the Youden index peaked at 0.425, with haemoglobin sensitivity to predict falls at 70.5% (the proportion of patients assessed as high‐risk among those who fell), and specificity at 72% (the proportion of patients identified as low‐risk among those who did not fall). The Youden index decreased with increasing haemoglobin levels, indicating that haemoglobin at 107.5 g/L is the optimal cut‐off point for predicting falls as it maximises the Youden index.

**TABLE 3:** Sensitivity, specificity, and Youden index at different haemoglobin.

Haemoglobin	Sensitivity	Specificity	Youden index
46–90	0–0.333	1–0.941	0–0.274
91.5–99.5	0.333–0.523	0.915–0.839	0.248–0.362
100.5	0.545	0.822	0.367
101.5	0.568	0.805	0.373
102.5	0.598	0.797	0.395
103.5	0.614	0.771	0.385
104.5	0.652	0.754	0.406
105.5	0.674	0.737	0.411
106.5	0.682	0.729	0.411
**107.5**	**0.705**	**0.72**	**0.425**
108.5	0.705	0.686	0.391
109.5	0.727	0.678	0.405
110.5	0.735	0.653	0.388
111.5	0.75	0.644	0.394
112.5	0.78	0.593	0.373
113.5	0.795	0.593	0.388
114.5	0.795	0.576	0.371
115.5	0.833	0.534	0.367
116.5	0.841	0.475	0.316
117.5	0.856	0.449	0.305
118.5	0.871	0.424	0.295
119.5	0.871	0.39	0.261
120.5	0.879	0.381	0.26
121.5–151	0.879–1	0.373–0	0.252–0

*Note:* Bold values indicate that at a haemoglobin level of 107.5g/L, the Youden index peaked at 0.425.

### 3.6 Characteristics of the Experts for the Delphi Surveys

Twenty‐one experts participated in each round of the consultation, including clinical nursing management experts, clinical nursing experts and clinical nursing teachers. They were all women and were from four hospitals in Zhejiang province in China, covering three municipalities. Table 4 shows the characteristics of the consultants in detail (*n* = 21).

**TABLE 4:** Characteristics of the consultants (*n* = 21).

Items	*N*	%
Education		
Undergraduate	18	85.71
Master	2	9.52
Doctor and above	1	4.76
Professional, years		
10–19	11	52.38
20–29	6	28.57
≥ 30	4	19.05
Professional qualifications		
Senior	5	23.81
Deputies senior	7	33.33
Medium	9	42.86
Position		
Director of nursing department	2	9.52
Nurse manager	13	61.90
Nurse sister	2	9.52
Clinical nurse	4	19.05

### 3.7 Expert Positive Factors and Authority Factor

There was a 100% recovery rate for the questionnaire. The expert's authority coefficient (Cr) was determined by three factors: the expert's academic level (q1), the expert's judgement basis for each item (q2) and the degree of familiarity (q3). The expert academic level weight assignment generally considered that the senior professional title was weighted as 1, the deputy senior professional title was weighted as 0.9 and the medium professional title was weighted as 0.8. Therefore, in this study, q1 = 0.881. The expert judgement was q2 = 0.805. The expert familiarity was q3 = 0.981. Hence, the Cr = (0.881 + 0.805 + 0.981)/3 = 0.889, indicating that the experts participating in this study were highly authoritative, and the consultation results were highly credible.

### 3.8 Level of Coordination of Expert Advice

The CV of expert advice in 5 rounds was 0.092–0.219, the CV below 0.15 reflecting good synerg. Table 5 shows the details.

**TABLE 5:** Level of coordination of expert advice.

Round	Theme	Minimum	Maximum	Mean	SD	CV
Round 1	Importance of anaemia in causing falls	4	5	4.62	0.498	0.108
	Haemoglobin as a screening factor for falls	2	5	4.43	0.87	0.196
Round 2	Importance of anaemia in causing falls	4	5	4.62	0.498	0.108
	Haemoglobin as a screening factor for falls	3	5	4.67	0.577	0.124
Round 3	The most reasonable stratification scheme	2	4	3.38	0.74	0.219
Round 4	The most reasonable stratification scheme	2	4	3.62	0.669	0.185
Round 5	Approvement of the final stratification scheme	4	5	4.76	0.436	0.092

### 3.9 The Consensus of Experts

After the first and second rounds of consultations, consensus on the importance of anaemia in causing falls and the use of haemoglobin as a screening factor for falls were obtained; 8 (38.1%) experts select an absolutely critical on anaemia in causing falls, and 13 (61.9%) experts select a fairly importance, respectively. The content validity index (I‐CVI) = 1, adjusted Kappa value (K*) was 1 and consensuses were reached by 20 (95.24%) experts: anaemia significantly contributes to falls in obstetrics and gynaecology wards, and haemoglobin should be employed as a screening factor for falls.

**FIGURE 2:**
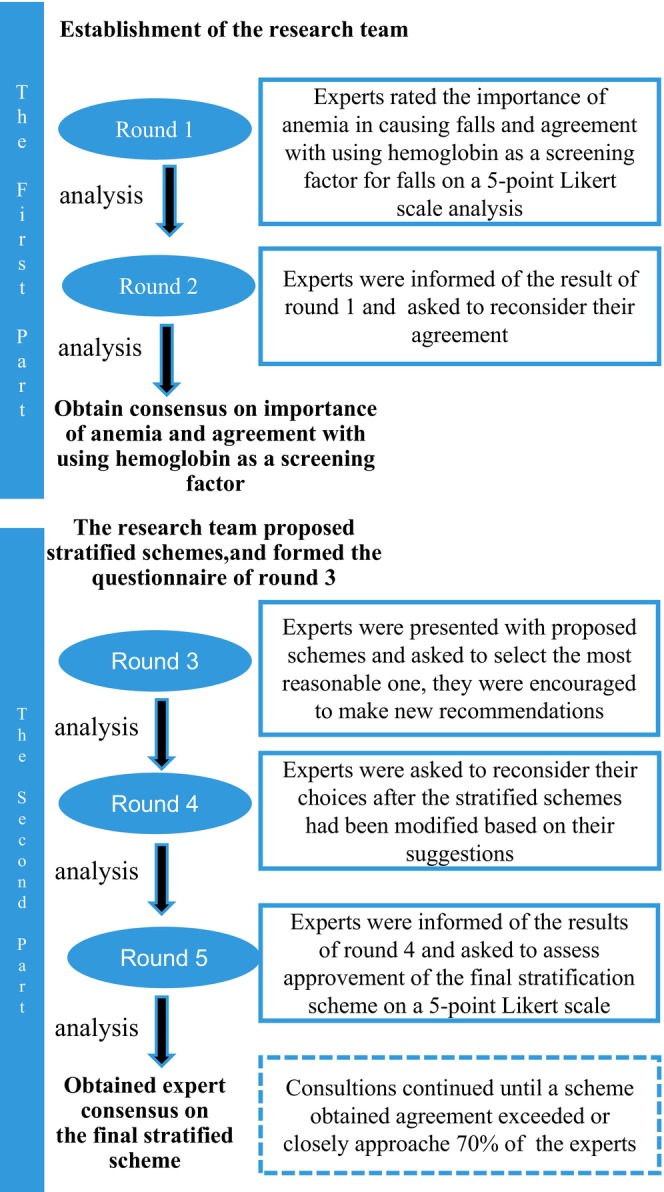
The process of the Delphi surveys.

The research team initially proposed five stratification schemes in the third round of consultation: (1) 90 g/L is divided into two levels, namely, ≥ 90 and < 90 g/L; (2) the 60 g/L boundary is divided into two levels, ≥ 60 and < 60 g/L; (3) with 90 and 60 g/L divided into three levels, ≥ 90, 60–89 and < 60 g/L; (4) according to the WHO obstetric anaemia criteria: ≥ 110, 100–109, 70–99, 40–69 and < 40 g/L; (5) according to the severity of the disease: ≥ 120, 90–119, 60–89 and < 60 g/L. The first and second schemes were eliminated due to the low selection rate, and 11 (52.38%) experts chose the fifth stratification scheme and proposed a modification that changed haemoglobin 120 to 110 g/L (CV = 0.189, > 0.15). Fourteen (66.67%) experts chose the fifth stratification scheme in the fourth round of consultation. The final scheme was determined after the fifth round of consultation, receiving agreement from experts. Sixteen (76.19%) experts strongly agreed, while five (23.81%) experts simply agreed with the final results. Haemoglobin for fall risk screening was stratified as follows: ≥ 110; 90–109; 60–89; and < 60 g/L. Figure 2 summarises the process in detail.

## 4 Discussion

This study investigates the potential relationship between anaemia and falls among obstetrics and gynaecology inpatients by retrospective survey and obtained a stratification scheme for haemoglobin as a screening tool for fall risk by Delphi surveys. This is one of the few reports of falls among hospitalised patients with Obstetrics and Gynaecology diseases.

The initial discovery indicates a fall rate of 0.21% (0.504 days per 1000 patient days) in obstetrics and gynaecology wards in china, contrasting lower than in rehabilitation and internal medicine wards (1.915% and 1.181%, respectively) (Mikos et al. 2021), similar with hospitalised adult patients with hematologic conditions were observed (Miwa et al. 2017). Notably, fall rates and trends vary significantly among clinical departments, and no serious injuries were reported, probably due to the unique patient characteristics, as reported by Mikos et al. (2021). Obstetrics and Gynaecology inpatients are generally under 65 years of age and in the reproductive stage, possessing better muscle strength and physiological functionality than their elder counterparts.

The second key finding highlights a correlation between surgery and falls, aligning with findings in other surgical wards, where post‐surgery dizziness, analgesics and postural hypotension contribute to fall risks (Hou et al. 2017). According to Kronzer et al. (2016), 24%–48% of preoperative patients fell in the 3–12 months before surgery. Seventy per cent of the participants suffered an injury. 0.8–16.3 falls per 1000 person‐days occurred after surgery, with a gradual decline in the months following. Approximately 10%–70% of fallers experienced some form of injury after a postoperative fall, and 5%–20% had severe injuries. We have therefore been reminded that fall prevention during surgery must be further investigated, as well as in Obstetrics and Gynaecology wards, inpatients undergoing surgical procedures should be carefully monitored by nurses to prevent falls.

Thirdly, our study establishes a link between lower haemoglobin levels and fall events in obstetrics and gynaecology inpatients during hospitalisation. The AUC of haemoglobin for inpatients of Obstetrics and gynaecology was 0.762, suggesting that haemoglobin has a good predictive value for falls among obstetrics and gynaecology inpatients. The conclusion aligns with the findings of Karl E. Steinberg, who reported a correlation between anaemia and falls especially as patients with lower haemoglobin levels are more likely to decline, which is not surprising to any clinician and nurse. The weaker a person is, the more likely she is to fall (Steinberg 2006).

Furthermore, the Delphi surveys provided confirmation that experts reached a consensus on the significance of anaemia in contributing to falls in obstetrics and gynaecology wards. They also agreed that haemoglobin should be employed as a screening factor for identifying fall risks. Recognising that the impact of anaemia on fall risks is contingent on haemoglobin levels, we implemented a stratification of haemoglobin, leading to the development of haemoglobin‐stratified schemes. As per the expert consensus, the stratification for haemoglobin in fall risk screening is as follows: ≥ 110; 90–109; 60–89; and < 60 g/L. A lower haemoglobin level indicates a higher risk grade for falls.

Additionally, we conducted an analysis to determine the optimal cut‐off point for anaemia to complement the stratification scheme. According to Li et al., the Youden Index is frequently employed to gauge the effectiveness of a diagnostic marker and facilitates the selection of an optimal threshold value (cut‐off point) for the marker. A large Youden index suggests that both sensitivity and specificity are relatively high, corresponding to lower misdiagnosis and missed diagnosis rates (Li et al. 2013). In this study, the Youden index reached its maximum at a haemoglobin level of 107.5 g/L, indicating that haemoglobin at this threshold is the optimal cut‐off point for predicting falls, with relatively low rates of misdiagnosis and missed diagnosis.

We considered both the Youden index and expert consensus. Since a haemoglobin level of 110 g/L is more practical for nurses in clinical application, and the Youden index ranges from 0.388 to 0.405, surpassing the values of the Hendrich II Fall Risk Model (0.35), Morse Fall Scale (0.24) and Johns Hopkins Fall Risk Assessment Tool (0.19) (Cho et al. 2020), we propose the following stratified scheme for haemoglobin in fall risk assessment for Obstetrics and Gynaecology patients: low fall risk (≥ 110 g/L), medium fall risk (90–109 g/L), high fall risk (60–89 g/L) and fairly high fall risk (< 60 g/L). When the inpatient's haemoglobin is < 110 g/L, nurses should be more vigilant about fall risk and implement preventive measures. Lower haemoglobin levels indicate a higher risk grade for falls.

In summary, this research highlights the importance of considering haemoglobin levels in fall risk assessments in Gynaecological and Obstetric Wards, with significant potential to improve both patient outcomes and clinical practices. For research, further studies are essential to validate these findings across diverse populations and settings, exploring the relationship between haemoglobin levels and fall risk in other wards or among patients with different clinical conditions. Additionally, integrating haemoglobin levels with other known fall risk factors, such as muscle weakness, balance issues or medication use, could lead to a more comprehensive risk assessment tool. For clinical practice, the proposed stratified anaemia scheme enables early identification and intervention for patients at higher risk of falls based on their haemoglobin levels, allowing for timely interventions like increased monitoring, tailored physiotherapy or nutritional support. Clinicians can also use this scheme to develop personalised care plans that address both anaemia and fall risk, ensuring that patients with haemoglobin levels below 60 g/L receive more intensive interventions compared to those with levels above 110 g/L.

### 4.1 Limitations

The study has several limitations. Firstly, the data were randomly selected, but the no‐fall group consisted of only 120 patients, preventing the analysis of fall rate variations across different haemoglobin levels based on patients' haemoglobin grades. Secondly, the conclusions were constrained by the retrospective design's limitations. The data were collected retrospectively, making it challenging to definitively determine the causes of inpatient falls. We recommend future research to be prospective in nature. Additionally, our study may be susceptible to confounding since patients deemed high risk for falls may have received fall prevention treatments, given the hospital's prioritisation of patient safety.

## 5 Conclusions

Globally, hospital safety and fall prevention are priorities, that's why the study was conducted. This study provided nurses with valuable information on screening fall risk in obstetrics and gynaecology patients, identifying the most critical risk factors and improving fall prediction accuracy. A stratified scheme for haemoglobin for fall risk assessment is also provided, providing nurses with a reference for their practice. In addition, nurses should closely observe inpatients in Obstetrics and Gynaecology wards who have Surgery and haemoglobin below 110 g/L, to provide appropriate fall prevention interventions.

## Author Contributions

**Yan Chen:** conceptualisation, methodology. **Yihan Ma, Chunsheng Wang:** data curation. **Ya Shen, Huiping Jiang:** original draft preparation. **Huifeng Gu:** visualisation, investigation. **Ya Shen:** supervision. **Luping Liu:** software, validation. **Bijun Mao**, **Peihong Zhou:** writing – reviewing and editing.

## Statistics

The statistics were checked prior to submission by Chunsheng Wang, he is an expert statistician.

## Conflicts of Interest

The authors declare no conflicts of interest.

## Data Availability

The data applied in this study is openly available: DOI 10.17605/OSF.IO/CM6WX; https://osf.io/cm6wx/. No use of generative AI and AI‐assisted technologies in the writing process.
